# ‘It’s my back…’; developing the coming to spinal clinic resource to improve the health literacy of young people with adolescent idiopathic scoliosis and their parents

**DOI:** 10.1177/13674935221083767

**Published:** 2022-04-18

**Authors:** Lucy Bray, Catherine Wilkinson, Colin Bruce, Neil Davidson, Candice Satchwell, Bernie Carter

**Affiliations:** 1 Faculty of Health Social Care and Medicine, 6249 Edge Hill University, Ormskirk, UK; 2 4593 Alder Hey Children’s NHS Foundation Trust, Liverpool, UK; 3 Centre for Excellence in Learning and Teaching (CELT), 6723 University of Central Lancashire, Preston, UK

**Keywords:** Young people, adolescent idiopathic scoliosis, health literacy, consultation, children’s participation

## Abstract

This study focussed on identifying the key concerns and information needs of young people with Adolescent Idiopathic Scoliosis (AIS) and their parents and examined what resources might help improve young people’s ‘participativeness’ and health literacy during clinic consultations. A qualitative participatory design underpinned the study. Workshops involving multiple methods were used to engage with young people with AIS and their parents, who were recruited through a regional children’s hospital. The study design was informed by patient and public consultation with eight young people and two parents. 10 young people (aged 14–16 years) and 11 of their parents participated in the study. Young people and their parents reported uncertainty and anxiety before coming to clinic and faced issues participating in the consultation, being involved in decision-making and understanding the information and language. These challenges resulted in unmet information needs. Young people’s health literacy relating to an AIS diagnosis and treatment is facilitated by them being prepared and informed *before* coming to clinic and be actively supported to be involved *during* the consultation. We collaboratively developed the ‘Coming to Spinal Clinic’ resource to help young people with AIS and parents prepare for and get the most out of their visit.

## Background

Adolescent Idiopathic Scoliosis (AIS) is the most common form of scoliosis (Cheng et al., 2015) and typically develops between the ages of 10 and 18 years (Altaf et al., 2013). Following diagnosis by a specialist, there are three common treatments: monitoring (young person’s curvature is reviewed at regular periods for progression), bracing (young person wears a brace to slow down the growth of curvature) or surgery (young person undergoes a surgical procedure to insert a metal rod to correct curvature) (Horne et al., 2014; Bettany-Saltikov et al., 2015).

Consultations with the specialist spinal team have been identified as young people’s and their parents main source of information following diagnosis (Bettany-Saltikov et al., 2015; van Shaik et al., 2007). Despite this, these consultations do not always address families’ information needs (Khetani et al., 2008; Bull & Grogan 2010; Rullander et al., 2013; MacCullloch et al., 2009) and young people can struggle to be meaningfully involved in these clinical interactions (Bettany-Saltikov et al, 2015). Sub-optimal consultation experiences can result in young people with AIS and their parents having poor levels of knowledge and understanding of their condition and the options for and implications of treatment (Bettany-Saltikov et al., 2012; van Shaik et al., 2007; Bull and Grogan 2010; Bray and Craske 2015; Wellburn et al., 2019).

Challenges linked to young people’s meaningful involvement in clinical consultations are well documented, with evidence highlighting how adult voices (parents and health professionals) can marginalise them during these encounters (van Staa and On Your Own Feet Research Group, 2011; Tates & Meeuwesen 2001; Gessler et al., 2019). Young people report a lack of voice in treatment decisions (Bray et al., 2012; Coyne et al., 2014), despite research which clearly demonstrates that young people value being part of these conversations (van Staa and On Your Own Feet Research Group, 2011). Actively involving young people in health consultations facilitates their ability to acquire information, gain an improved understanding of their condition and health care, increases their ability to communicate choices and decisions and enhances their health literacy (Gessler et al., 2019; Nutbeam 2008; Rubin et al., 2011). Health literacy is defined as ‘the cognitive and social skills which determine the motivation and ability of individuals to gain access to, understand and use information in ways which promote and maintain good health’ (Nutbeam 2000, pg264). Consultations and interactions with health professionals are identified as a key way for people to access credible information, enhance their understanding of health information (Paasche-Orlow & Wolf 2007; Bray et al., 2019) and participate more actively and confidently in decision-making (Nutbeam 2008). ‘Participativeness’ in health encounters (Rubin et al., 2011; Baker 2006) is linked to patient empowerment. Improving patients’ ability to communicate with their health providers, increases their satisfaction with health professionals and services (Trumble et al., 2006), reduces their anxiety (Roter and Hall, 2006) and improves their understanding of their condition and adherence to treatment regimens (Nutbeam 2000; Peerson & Saunders 2009; Ishikawa & Kiuchi 2010). ‘Participativeness’ is often linked with the term ‘interactive health literacy’, which is defined as the ‘proclivity of patients to participate actively in interpersonal health information exchange’ (Rubin et al., 2011, pg 3).

When we scoped the literature, there was evidence of the development of materials and resources to facilitate the involvement of young people in consultations relating to neurology (Cunningham and Newton 2000), asthma (Milnes et al., 2013) and diabetes (Robling et al., 2012), but none relating to scoliosis or spinal consultations. This study aimed to address the lack of resources to facilitate young peoples' involvement in spinal clinic.

### Aim

To identify the key concerns and information needs of young people with AIS and their parents when attending spinal clinic and examine what resources or interventions might help improve young people’s ‘participativeness’ and heath literacy during clinic consultations.

## Research design

A qualitative participatory approach involving multiple methods and activities was used to engage meaningfully and collaboratively with young people and their parents. This design underpinned our core values of working *with* and alongside young people and their parents in a democratic, collaborative and supportive way (Bergold & Thomas 2012; Haijes & Van Thiel 2016).

### Patient and public involvement and engagement

During the development of this study, we consulted with eight young people and young adults with AIS and two parents of young people with AIS. Young people and parents were contacted through liaison with the Scoliosis Association UK (SAUK). Patient and Public Involvement and Engagement (PPIE) consultation occurred via the telephone and focussed on attending spinal clinic and the role of spinal consultations in families gaining credible information. The young people and parents discussed feeling uncertain about what questions to ask during spinal consultations and that this had resulted in them having unmet information needs. The young people felt that the focus of the study on developing an intervention to help interactions in clinic would be useful. Ongoing feedback from four of these young people with AIS and a parent informed the development of our study information documents and plans for our data collection workshops. In line with best practice, we have reported this consultation according to the statements in the GRIPP2 (Guidance for Reporting Involvement of Patients and the Public) short form (Staniszewska et al., 2017).

## Research methods

Two separate participatory workshops with similar structures and aims were undertaken concurrently with young people with AIS (facilitated by LB) and their parents (facilitated by BC and CW). The researchers facilitating the workshops were not associated with the clinical care of the young people with AIS. Separate workshops aimed to encourage freedom of expression with their peers before sharing any ideas with the wider group. Our workshops aimed to facilitate participant engagement, stimulate discussion and generate insights (Ruland et al., 2008; Iversen and Dindler 2014), into young people’s and parents’ information needs and the key concerns when attending spinal clinic. Workshops lasted 2 hours and were held in small meeting rooms in the same hospital the young people and their parents had previously attended.

Workshops were structured around three activities to generate qualitative data. The first activity used emojis and large flip charts to explore the feelings and emotions which may be associated with coming to spinal clinic. Young people and parents were asked to select from laminated emoji images or draw their own emoji to add to the flip charts to represent these feelings and emotions. Participants were then asked to examine and explain why a person attending clinic may experience these emotions. The second activity examined information needs, and speech bubbles were used to prompt young people and parents to ‘write and tell’ (Noonan et al., 2016) and think about what may be important questions to ask in clinic. This activity enabled the researchers to explore the experiences of seeking information within a clinic appointment and the prompts and key phrases which may facilitate interactions with health professionals. The third activity used thought clouds to prompt the young people and parents to think about what their ‘top tips’ would be to other young people and parents attending spinal clinic for the first time. Young people and parents were asked to write their ideas on the paper clouds and create a ‘top tips’ collection. This activity prompted the group to share both positive and challenging aspects of consultations and the lessons they had learnt through their prolonged engagement.

There was opportunity during and outside of the workshop activities for young people and their parents to raise additional issues, concerns and experiences about attending clinic that they did not wish to share with other participants. Workshops were audio-recorded to capture responses, preferences and discussions. Activity sheets and written work were photographed and field notes were recorded throughout the workshops by the researchers.

### Sample and participant recruitment

Eligible young people were 13–18 years of age, had completed their post-operative clinical journey or had undertaken up to 12 months of conservative treatment (bracing) and be able to communicate using English. Young people and their parents were identified and initially approached to participate in the study via an information pack posted to them by their clinical team containing a parent information leaflet and a young person’s information leaflet. The young person and their parent(s) had at least 2 weeks to decide whether they would like to take part. Parents could participate independently from their child, and young people could take part even if their parent did not wish to participate. If the parent and/or young person were interested in taking part, they were directed on the information leaflet to contact the research team (via email, text or telephone to designated project phone). A member of the research team then contacted them via the telephone to check they had read the information leaflet, talk through the project and answer any questions.

### Ethical considerations

Ethics approval was granted by the authors’ institution and the Health Research Authority (17/LO/0261). Before the workshop activities commenced key ‘ground-rules’ were established, for example, respecting people’s opinions and not sharing information about other people outside the group. Written consent was obtained from parents (for their own involvement and involvement of children <16 years of age). Young people aged <16 years gave written assent and those aged 16 or over gave written consent. Participants were clear that once they had participated in the workshop they would not be able to withdraw their data, as it would be too difficult to separate out their individual contribution.

One of the research team members (CW) was available to support participants if they became upset, needed to leave the workshop or take a break, although this support was not required. At the end of the workshop a researcher checked that the participants were okay and signposted them, if appropriate, to the national scoliosis charity SAUK.

### Analysis

Data from the workshops were primarily qualitative (notes from activities, activity sheets, visual images, transcripts of discussions and reflexive field notes). Some preliminary data analysis occurred within the workshops as the young people and parents discussed and decided on ‘themes of importance’ and what was not relevant. However, after the workshops, the data were subjected to more theoretical analysis using a thematic approach (Braun &an Clarke 2006). Thematic analysis was conducted by all members of the research team (LB, BC and CW), who engaged in the inductive and iterative process of constructing codes and themes. The analysis examined any similarities and contrasts between the perspectives of the young people and their parents.

### Findings

The workshops involved 10 young people (aged fourteen–sixteen years, nine girls and one boy, eight with surgical correction, two with a brace) and 11 of their parents. The interactional nature of the activities meant that it was sometimes difficult to identify individuals from the recordings so none of the quotes are linked to specific individuals. The findings are presented under the two themes of ‘*Into the unknown: uncertainties and expectations associated with coming to spinal clinic*’ and *‘I didn’t understand and didn’t know what to ask: information seeking during spinal clinic’*. Both themes are linked to young people’s ‘participativeness’ (active engagement in meaningful information exchange in order to be involved in decisions about their scoliosis). Findings demonstrate that supporting young people’s health literacy relating to an AIS diagnosis and treatment requires them to be prepared and informed *before* coming to clinic and be supported to be involved *during* the appointment.

#### ‘Into the unknown’: Uncertainties and expectations associated with coming to spinal clinic

Young people described how coming to spinal clinic for the first time was *‘a big deal’* for them and many had felt *‘scared and worried’, ‘uncertain’* and *‘overwhelmed’.* These feelings were often linked to *‘not knowing what was going to happen’* or what to expect *‘coming to a big hospital for the first time’*. Many had been told about AIS before coming to clinic to see a specialist and had felt *‘confused’* and *‘shocked as suddenly you are told you have this condition that you have never heard about’*. In contrast, some of the young people described feeling happy attending clinic as it meant that they were seeing *‘someone to help them find a solution’* and it was hopefully the beginning of a journey to *‘get [back] straight again’*.

Parents also said they had felt *‘worried, anxious, and frightened’* coming to clinic for the first time as it felt like ‘*going into the unknown’* as they ‘*did not know what to expect’*. Some also discussed how they had felt guilty that they had not noticed the curve earlier and how they were ‘*relieved to finally be at the appointment*’ with the hope of getting a clear idea of treatment with *‘the chance to ask questions’*.

Many of the parents, who had been told a tentative diagnosis of scoliosis from primary care services, had ‘Googled’ it before coming to spinal clinic. This internet searching had, in some cases, been helpful showing ‘*what our X ray rooms looks like, there’s the X ray, you’ll go in, your parents can stand here*’ or outlining possible treatment paths. For some young people, searching the internet or social media had caused them shock and worry, ‘*I was absolutely terrified by what I saw as actual pictures of the surgery came up’*. The young people had struggled to find any information from other young people and felt that this would have been *‘the most useful thing’* as *‘having information from other young people is really important’* and ‘*helps you worry less’.* None of the participants had accessed resources which focussed on what would happen during a spinal clinic appointment or how to prepare for such an appointment. Overall, young people felt that a resource to prepare other young people coming to spinal clinic would be useful and should look engaging, be ‘*bright and interesting’* and be clear it was ‘*developed with young people’.*

#### ‘I didn’t understand and didn’t know what to ask’: Information seeking during spinal clinic

During clinic, the young people discussed feeling ‘*shocked and surprised*’ when they saw their spinal X-ray for the first time and at how large their curve was and expressed ‘*worry about the treatment to come’*, which for eight of them had been surgery. When professionals directed the conversation towards the young people, this had helped them feel valued and recognised and they appreciated being ‘*told everything that was going to happen’*. They also appreciated conversations where clinicians were *‘not just talking to my mum but talking to me as well and including me’*. Despite being talked to directly, young people explained it could be ‘*hard to listen when you have just been told*’ particularly as ‘*everything doesn’t make sense… you are not really listening to it or taking it in’.* This resulted in them being part of the consultation, although they did not always ‘*understand everything being discussed*’ as the clinicians used *‘words that do not always make sense’*.

Some young people identified how it could be difficult to actively ask questions in clinic as they either ‘*didn’t know what to ask*’ or ‘*how to start to join in’*. Some young people reported that this limited their involvement with one young person noting *‘I said two words like the whole time’* and another talking about *‘feeling more worried’*. Some young people described the consultation as being dominated by their parents who had their own questions and ‘*seemed to know what to ask and what was going on*’. Whilst some young people talked of their parents taking the lead being a good thing as *‘I just wanted to listen and not know too much’*, others felt it was important for everyone to *‘remember you are the one going through it, not your parents*’.

All young people were keen to feel empowered to *‘not be afraid to ask’* their own questions at clinic ‘*to find out what is happening’.* They thought that preparing possible questions before their appointment *‘would help*.’ Also, they agreed that it would be useful if the questions were on something tangible this would *‘spark your memory if you got stuck*’. Many had felt more able to ask questions on their second or third appointment when they had *‘kind of settled in and it has sunk in a bit more and you know what is going on’*. The young people discussed questions they wished they had asked at clinic and ones they thought were important for other young people, including ‘*what caused my scoliosis*?’ and *‘what are the different types of treatment*?’ (see Table 1). The young people also discussed how ‘*everyone will feel something different coming to clinic and will want to know different stuff’*, and that it was important for young people to have their own dedicated information and materials to empower them to engage in clinic conversations; appointments were identified as *‘really important sources of information’*.

**Table 1.** Categories of information needs of young people and parents when attending spinal clinic.

Aetiology-related questions	
Young person	Parent
What caused my scoliosis?	What has caused it?
What is scoliosis?	Will my other children be okay?
Why do I have it?	How far might the curve/s progress? How bad could it get (the curve)?
How bad is my curve?	What part of the spine is it?’
	What degree is the curve?
	What are the possible outcomes?
Treatment-related questions	
Young person	Parent
What are the different types of treatment?	What are the treatment options/plan?
What does each treatment involve?	What are the risks of surgery?
How will it impact on my life?	What are the recovery times of surgery?
Which is the best treatment for me?	At what stage is surgery necessary?
What happens during surgery?	Are there any alternative therapies?
Is surgery my only option to look normal?	
Will I be able to do everything I already do after surgery?	
Will it cause me any pain in the future?	
What will I be able to do after surgery?	
Is this hospital a good place to have surgery?	
Are there other options outside of the NHS?	
Support-related questions	
Young person	Parent
Where is an appropriate website to find out more information for my age?	Who do we contact with any extra questions?
Who/where should I go for help and advice? Websites? People?	What will happen at the next appointment?
What can I do to help?	Where is a good place to look for more information?
What about my body image?	How often will we see the consultant?

Many of the parents said the consultations were key for gaining information but had struggled to feel able to ask questions, with some parents coming away from clinic with ‘*lots of unanswered questions*’. A few parents had ‘*jotted [questions] down on a notepad*’ to help them *‘remember what to ask’* and gain information during the consultation. Questions parents identified as important were similar to those of their children with a focus on causes of scoliosis and treatment options (detailed in Table 1), such as ‘*What are the treatment options/plan?*’ and ‘*At what stage is surgery necessary?*’ Although parents discussed all the questions they had, many also recognised that their child would have their own questions and concerns and how it could be challenging to, ‘*let your child ask questions*’ as well.

The young people and parents all felt that a question prompt sheet or ‘prep’ sheet would help them *‘feel able to ask questions in clinic’* and a physical copy would help ‘*make sure you could write down what they have said’* and this would help them *‘remember what was talked about when you get home’.*

### The ‘Coming to Spinal Clinic’ resource

**Figure 1.**
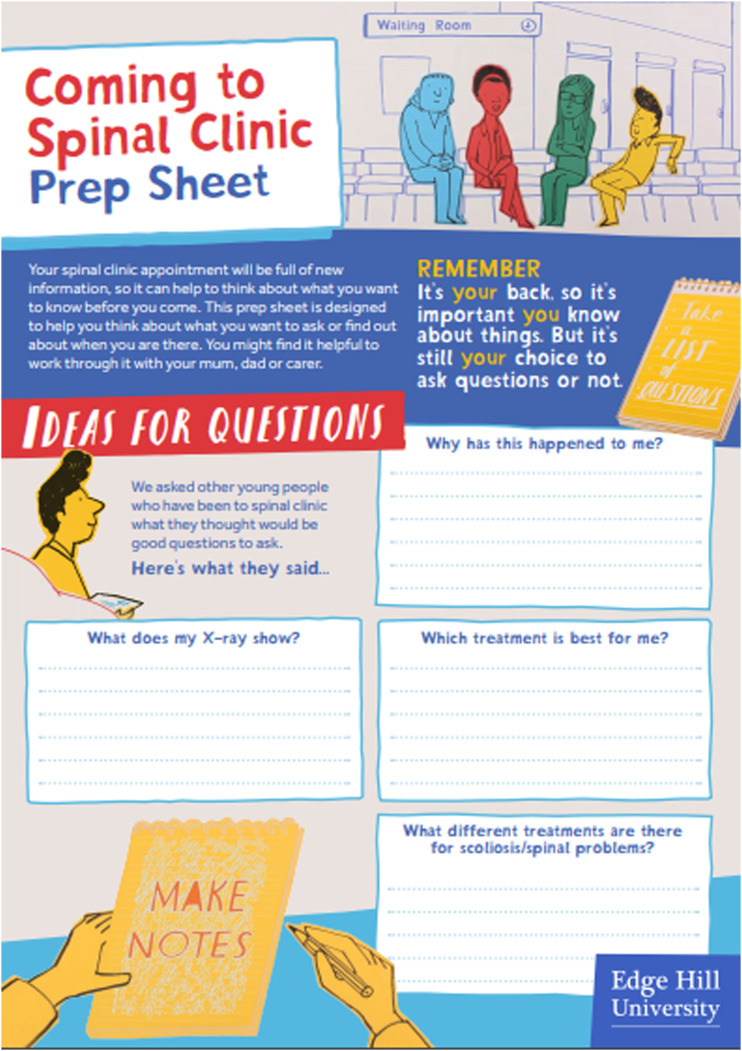
Prep sheet for young people ‘Coming to Spinal Clinic’ 264×374 mm (38 × 38 DPI).

**Figure 2.**
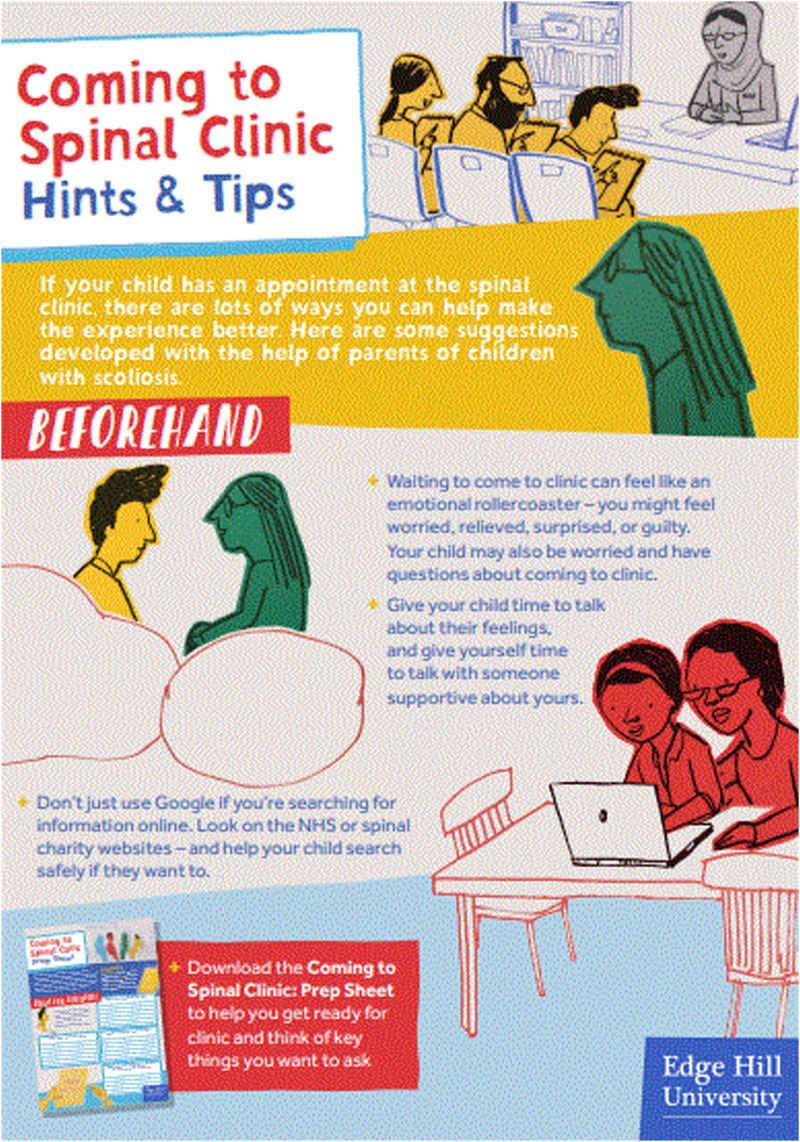
Parent information for ‘Coming to Spinal Clinic’ 136×195 mm (72 × 72 DPI).

We iteratively developed the Coming to Spinal Clinic resource (link to be inserted after peer review) through a process of ongoing consultation with young people with AIS and their parents, based on their identified information needs. This resource comprises (1) an animation for young people, (2) a ‘prep’ (prompt) sheet with top tips to empower young people to be able to craft and ask questions in clinic about their condition and possible treatment path (Figure 1) and (3) an information sheet for parents to help prepare them for coming to spinal clinic and to reduce their feelings of entering ‘into the unknown’ (Figure 2). The resource is free to use and download and is currently accessible via various websites (university, hospital and key spinal charities).

## Discussion

This study examined the key concerns and information needs of young people with AIS and their parents when attending spinal clinic and highlighted how important these consultations are as a source of information. Despite the importance of consultations, the context of these visits often challenged aspects of young people’s health literacy; their ability to access, understand and use information to make health-related decisions (Nutbeam, 2008). This in turn could challenge young people in feeling empowered (Coyne 2008) and functioning effectively within the health system (Edwards et al., 2015). Young people with AIS and their parents reported in this study that they were worried and anxious and did not know what to expect when they came to spinal clinic. Young people particularly discussed how they struggled to be meaningfully involved in information exchange during these consultations and this could lead to unmet information needs. This study highlights that for young people’s ‘participativeness’ and developing health literacy to be facilitated they require information and guidance before a health care consultation, as well as interventions to support them during a clinic visit.

As part of this study, we recognised the value in working with young people to develop a resource to address the issues encountered in spinal clinic, which focussed on facilitating young people to improve their health literacy. The young people identified that a prompt sheet would help them identify, craft and raise questions during a consultation in order to gain and interpret individualised information. The young people preferred the use of the term ‘prep sheet’. The use of professionally endorsed prompt sheets is linked to higher levels of patient involvement and significantly more information being provided by a doctor during consultations (Sansoni et al., 2015); such interventions are mainly reported within adult health care contexts, particularly within cancer care settings (Bruera et al., 2003; Dimoska et al. 2012). In relation to health literacy, prompt sheets help people feel empowered to ask questions of importance to them, which helps them individualise and process health information, gain an increased understanding and have improved health status (Roter and Hall, 2006). The development and use of interventions to facilitate involvement of children and young people in health care consultations is far less evident (Bray et al., 2018). However, studies have shown that prompt sheets can help young people with endocrine conditions influence consultation agendas and raise questions (Downing et al., 2017) and highlight issues which otherwise may go unaddressed (Cunningham and Newton 2000). These previous studies focus on children and young people with long-term conditions and there is less work which has focussed on interventions to facilitate young people with diagnoses made within adolescence or with minimal health care experience.

This lack of health care experience or ‘know how’ was raised by the young people, who identified that their engagement during a consultation could be facilitated by information to help them prepare for coming to spinal clinic, to know what to expect and anticipate how they may feel. Such preparatory information can help empower young people through familiarising them with what will happen when they come to hospital and lay the foundations that their opinions and active involvement are encouraged (Bray et al., 2019). A clinic visit can disempower young people, with the high levels of uncertainty, short nature of many appointments, wealth of information exchange, adult agendas and unfamiliar people, terms and processes (van Staa and On Your Own Feet Research Group, 2011). This can be particularly pronounced for young people who have minimal health care experience and are dealing with a new diagnosis (Bray et al., 2012) and who struggle to take on board information in this unfamiliar environment (Dovey-Pearce et al., 2005). The expanded models of health literacy acknowledge the importance of context on a person’s health literacy, the influence of situational factors, social relations and structures on a person’s ability to access, understand and use information to make meaningful decisions (Nutbeam 2000; Chinn 2011; Samerski 2019). If young people are informed about what a clinic visit involves and how to prepare for a consultation *before* arriving at hospital, we propose they will be more able to participate meaningfully during the information exchange.

Our focus on the needs of young people was not intended to underplay the important role parents play in supporting their child’s journey through AIS diagnosis and treatment and how parents contribute to and support their child’s access to information and involvement in consultations (Bettany-Saltikov et al., 2012; Wellburn et al., 2019). Parents have an important role in facilitating their child’s acquisition of health literacy, which is recognised as a life-long process starting in childhood (Broder et al. 2017). There is increasing acknowledgement that health literacy can be ‘distributed’ through immediate social and familial circles (Edwards et al., 2015; Bray et al., 2021). Family members can be ‘literacy mediators’ a term currently only used in reference to adult family members, but also applicable to young people and their parents and one which resonates with other literature describing parents as ‘communication buffers’ (Coyne et al., 2016) and ‘information filters’ (Lambert et al., 2013).

This study has highlighted that parents have their own distinct information needs and can themselves experience difficulties in understanding information within a specialist spinal consultation. This reflects other evidence from a recent systematic review which highlights how parents whose child is diagnosed with AIS can find clinic attendance stressful and can struggle to acquire credible information to join in decision-making (Motyer et al., 2020). Motyer et al. (2020) note that parents need support in gaining information to help reduce their anxiety and emotional burden when their child is diagnosed with AIS and requires surgery. Our findings reveal that parents can have distinct and differing information needs to their child, which demonstrates a need for dedicated information resources. Therefore, despite our original intention to solely develop resources for young people, the parents involved in this study identified a need for their own bespoke information to help them support their child.

This study has demonstrated that spinal consultations involve high levels of information exchange and are reported as complex; young people and their parents identify that their health literacy would be facilitated by interventions to help them prepare and engage effectively for a spinal consultation.

## Study limitations

The study used a small convenience sample of young people with AIS and their parents from one hospital trust, and participants’ views may not represent those of other young people with AIS and their parents elsewhere. This small study collaboratively developed a resource with young people with AIS with the aim of improving their participation in spinal consultations, there is therefore further work needed to evaluate the resource in terms of its acceptability and impact within a clinical setting.

### Implications for practice

This study highlights that young people with AIS and their parents require information and guidance before a health care consultation to help them prepare for the clinic visit. Study findings also highlight that health professionals need to actively facilitate young people and their parents’ involvement and participation in a spinal consultation. Being able to participate meaningfully in a spinal consultation is vital for young people and parents to gain credible information and make meaningful treatment and management decisions. We propose the collaboratively developed ‘Coming to Spinal Clinic’ resource has the potential to provide some structure to help guide the preparation for and discussion during a spinal consultation.

## Conclusion

Young people with AIS and their parents reported uncertainty and anxiety before coming to spinal clinic and could face challenges during consultations in being involved in information exchange due to the complexity of the language used and difficulty in meaningfully participating in the discussion. Despite spinal clinics being an important source of credible information for young people with AIS and their parents, the challenges to ‘participativeness’ in clinic resulted in unmet information needs. Young people’s health literacy relating to an AIS diagnosis and treatment is facilitated by them being prepared and informed *before* coming to clinic and be actively supported to be involved *during* the consultation.
